# Validation of a Quantification Method for Curcumin Derivatives and Their Hepatoprotective Effects on Nonalcoholic Fatty Liver Disease

**DOI:** 10.3390/cimb44010029

**Published:** 2022-01-15

**Authors:** Young-Seob Lee, Seon Min Oh, Qian-Qian Li, Kwan-Woo Kim, Dahye Yoon, Min-Ho Lee, Dong-Yeul Kwon, Ok-Hwa Kang, Dae Young Lee

**Affiliations:** 1 Department of Herbal Crop Research, National Institute of Horticultural and Herbal Science, RDA, Eumseong 27709, Korea; youngseoblee@korea.kr (Y.-S.L.); seonmin88@korea.kr (S.M.O.); swamp1@korea.kr (K.-W.K.); dahyeyoon@korea.kr (D.Y.); 2 College of Pharmacy and Wonkwang-Oriental Medicines Research Institute, Institute of Biotechnology, Wonkwang University, Iksan 54538, Korea; aliceqql@163.com (Q.-Q.L.); sssimi@wku.ac.kr (D.-Y.K.); 3 Department of Food Technology and Services, Eulji University, Seongnam 11759, Korea; minho@eulji.ac.kr

**Keywords:** nonalcoholic fatty liver disease, *Curcuma longa* L., curcumin derivatives, quantitative analysis, HPLC, method validation

## Abstract

Curcumin (CM), demethoxycurcumin (DMC), and bisdemethoxycurcumin (BDMC) are major curcumin derivatives found in the rhizome of turmeric (*Curcuma longa* L.), and have yielded impressive properties to halt various diseases. In the present study, we carried out a method validation for curcumin derivatives and analyzed the contents simultaneously using HPLC with UV detection. For validation, HPLC was used to estimate linearity, range, specificity, accuracy, precision, limit of detection (LOD), and limit of quantification (LOQ). Results showed a high linearity of the calibration curve, with a coefficient of correlation (R^2^) for CM, DMC, and BDMC of 0.9999, 0.9999, and 0.9997, respectively. The LOD values for CM, DMC, and BDMC were 1.16, 1.03, and 2.53 ng/μL and LOQ values were 3.50, 3.11, and 7.67 ng/μL, respectively. Moreover, to evaluate the ability of curcumin derivatives to reduce liver lipogenesis and compare curcumin derivatives’ therapeutic effects, a HepG2 cell model was established to analyze their hepatoprotective properties. Regarding the in vivo study, we investigated the effect of DMC, CM, and BDMC on nonalcoholic fatty liver disease (NAFLD) caused by a methionine choline deficient (MCD)-diet in the C57BL/6J mice model. From the in vitro and in vivo results, curcumin derivatives alleviated MCD-diet-induced lipid accumulation as well as high triglyceride (TG) and total cholesterol (TC) levels, and the protein and gene expression of the transcription factors related to liver adipogenesis were suppressed. Furthermore, in MCD-diet mice, curcumin derivatives suppressed the upregulation of toll-like receptors (TLRs) and the production of pro-inflammatory cytokines. In conclusion, our findings indicated that all of the three curcuminoids exerted a hepatoprotective effect in the HepG2 cell model and the MCD-diet-induced NAFLD model, suggesting a potential for curcuminoids derived from turmeric as novel therapeutic agents for NAFLD.

## 1. Introduction

Recently, a variety of food and health supplements using natural substances have been distributed in Korea and many other countries [1,2,3]. Natural materials have fewer side effects and are relatively safe for mid- or long-term use based on long-term clinical experiences, so the increase in consumption and the expansion of the market are becoming more active [4]. Therefore, the standardization process is most important when developing and producing health functioning foods. Although natural food materials have secured their efficacy and stability through long-eating experience, differences in quality exist due to various variables such as soil, collection time, and cultivation conditions. In addition, there may be differences in the content of the major constituents in the process of heating and extracting them. Thus, it is essential to check the content of the surface ingredients and ensure that the content is validated and an analysis method considered to be accurate is applied to make the analysis more feasible and reliable [5,6,7].

Nonalcoholic fatty liver disease (NAFLD) is the most common liver disease globally, and, due to its increasing incidence, NAFLD has become a major health burden [8]. NAFLD is conceptualized as a hepatic manifestation of the metabolic syndrome and includes a range of conditions, from simple nonalcoholic fatty liver (NAFL) to the more severe nonalcoholic steatohepatitis (NASH), which may progress to cirrhosis and hepatocellular carcinoma [9]. Generally, simple steatosis is not lethal, while severe steatosis without concurrent inflammation may cause metabolic disorders [10]. Recent evidence suggests that NAFL occurs in nearly 85–95% of morbidly obese patients, with more than 30% of NAFL patients further progressing to NASH, in which steatosis coexists with inflammation and fibrosis, and may then progress to cirrhosis or even hepatocellular carcinoma [11]. Although the pathogenesis of NAFLD remains to be elucidated, many hypotheses have been proposed [12]. One of the widely accepted theories is the “two-hit theory”. The first hit refers to the accumulation of hepatic lipids, especially triglycerides (TGs), causing insulin resistance (IR) [13]. On this basis, oxidative stress and lipid peroxidation damage may be the major factors in the second hit, resulting in inflammation, necrosis, and fibrosis in the fatty liver [14]. Despite the universality of NAFLD, effective therapeutic drugs continue to be insufficient. Therefore, it is necessary to continue research on the pharmacological treatment of NAFLD [15].

A hallmark of NAFLD is excess TG accumulation within hepatocytes, and free fatty acids (FFAs) have been considered to play a predominant role in the pathogenesis and development of NAFLD [16]. Ultimately, an imbalance between FFA assimilation and disposal triggers steatosis, steatohepatitis, IR, and inflammation [17]. The sterol regulatory element-binding protein (SREBP) family has three isoforms, namely, SREBP-1a, SREBP-1c, and SREBP-2, which are important in the process of lipogenesis and cholesterogenesis [18]. In particular, SREBP-1c is a pivotal transcriptional regulator for the synthesis, oxidation, and transport of FFAs and TGs [19]. Adenosine monophosphate-activated protein kinase (AMPK), an obligate heterotrimeric complex composed of a catalytic (α) subunit and two regulatory (β and γ) subunits, is considered to be a critical functional enzyme for the management of hepatic steatosis [20]. Emerging evidence suggests that the activation of AMPK appears to have a protective effect on many diseases by adjusting the immune system and energy metabolism [21]. In addition, AMPK was reported to play an important role in providing metabolic adaptations as an important energy-sensitive protein [22]. AMPK is considered as a potential target for NAFLD treatment due to its capacity to inhibit lipid accumulation in adipocytes [23].

Traditional medicine has entered mainstream society and culture, and natural herbs have been used to treat various conditions for many years [24]. At present, there are few synthetic drugs with effective liver protection; therefore, it is a highly important task to investigate natural herbal extracts with hepatoprotective effects [25]. Turmeric, a perennial herb indigenous to India, is a well-known yellow colorant, spice, dying agent for clothing, and traditional medicine, and is obtained from the rhizome of *Curcuma longa* L. (*C. longa*) (Zingiberaceae) [26,27,28]. Turmeric has been widely distributed in tropical and subtropical regions of Southeast and Southern Asia [29]. The extract of turmeric has traditionally been used as a therapeutic method for its antioxidant, anti-migraine, anti-inflammatory, anti-tumor, and antimicrobial properties [30,31]. Turmeric is composed of compounds including diarylhepanoid, diarylpentanoid, phenylpropanoid, monoterpene, sesquiterpene, triterpene, and alkaloids [32]. The main component of turmeric is about 70% carbohydrates, and it is composed of curcuminoids, essential oils, proteins, polypeptides, and minerals [33]. Curcuminoids contain approximately 71.5% curcumin (CM), 19.4% demethoxycurcumin (DMC), and 9.1% bisdemethoxycurcumin (BDMC) as major curcumin derivatives (curcuminoids), which are all diarylheptanoids [34,35]. Among these, CM is a yellow pigment and a polyphenol, and is a well-known major compound of curcuminoids [36]. Extensive research has indicated that CM possesses a wide range of biological functions, such as anti-tumor, anti-metastasis, anti-angiogenesis, anti-inflammatory, anti-cancer, anti-oxidant, anti-malarial, and anti-microbial properties [37,38,39,40]. DMC, a chemical analog of CM, lacks the methoxy group on the benzene ring, which leads to a more stable structure [41]. DMC is known to have multiple biological characteristics, such as anti-tumor, anti-cancer, anti-oxidant, and anti-inflammatory activities [42,43]. BDMC is a polyphenolic compound with a diarylheptanoid skeleton, and is more stable than CM in a physiological medium [44,45]. BDMC shows various pharmacological activities, including anti-tumor, anti-apoptosis, anti-oxidative, and anti-inflammatory effects [46,47]. Since the discovery of the pharmaceutical properties of naturally occurring phenolic curcuminoids derived from turmeric, its potential medicinal values have been widely recognized in terms of health and nutrition [48].

By studying the effect of curcumin on high-fat and high-fructose diet (HFHFr)-induced NAFLD in C57BL/6 mice and mice hepatocytes, it has been reported that curcumin may prevent and treat NAFLD by targeting the liver X receptor-α (LXRα), which can transcriptionally regulate fatty acid biosynthesis [49]. Our prior in vivo research on BDMC has found its potential to inhibit MCD-diet-induced NAFLD in C57BL/6J mice and the synergistic effect between BDMC and silymarin, which is widely used as liver protective agent against hepatotoxicity [45]. In the present study, we establish an optimal analysis method using HPLC with a UV detector for turmeric. Not only was the validation of linearity, range, specificity, accuracy, precision, limit of detection (LOD), and limit of quantification (LOQ) performed, and the content of CM, DMC and BDMC investigated, but in vitro and in vivo experiments were also carried out to perform a comprehensive analysis of the anti-lipogenesis capacity of the three major curcuminoids derived from turmeric, and, consequently, to compare their effects on ameliorating NAFLD.

## 2. Materials and Methods

### 2.1. Preparation of Turmeric

Turmeric *(C*. *longa*) was harvested from the Jindo county, Jeonnam province, Korea. A voucher specimen (MPS00) was deposited at the Herbarium of the Department of Herbal Crop Research, National Institute of Horticultural and Herbal Science, Rural Development Administration, Eumseong, South Korea. The rhizome of the turmeric was coarsely ground and extracted twice by reflux extraction with 50% fermented ethanol at 80 °C for 4 h. After the extraction, extracts were filtered using a filter paper (No.1, WHATMAN, Maidstone, UK) and using a decompression distillation apparatus (N-1000, EYELA, Tokyo, Japan). The concentrated extract was obtained using a −80 °C ultra-low temperature freezer (DF8520, IlshinBioBase, Dongducheon, Korea) and freeze dryer (FDU-1200, EYELA, Tokyo, Japan). Standard substances were diluted with 100% methanol according to specific concentration and used for quantitative analyses and method validation. In addition, a fine powder of turmeric was extracted ultrasonically with methanol at 30 °C for 1 h.

### 2.2. Analysis of Turmeric Using HPLC

High performance liquid chromatography (HPLC) was performed using an Agilent Technologies HPLC 1200 series system (Agilent Technologies, Santa Clara, CA, USA) with YMC-pack ODS-AM (250 × 4.6 mm, 5 μm; YMC, Shimogyo-ku, Kyoto, Japan) for chromatographic separation. Column temperature was maintained at 35 °C and the absorbance at the detector was measured at 425 nm. The mobile phase was composed of water containing 0.1% formic acid (*v*/*v*, solvent A) and acetonitrile (*v*/*v*, solvent B) and was pumped at a flow rate of 1 mL/min. A 10 μL aliquot was injected into the column using the auto-sampler. The gradient elution conditions were set as follows: 0–15 min, B 50%.

### 2.3. Method Validation

CM, DMC, and BDMC from turmeric were dissolved in 100% methanol (MeOH) to create the standard quantitation curve. The three compounds were calculated for a regression curve and the linearity using HPLC was verified. The lowest detection limit concentration (LOD) and the lowest quantification limit concentration (LOQ) for the three compounds were obtained from the S/N value, which is the noise (N) relative to the signal (S), and automatically calculated; the S/N values were 3 and 10. In addition, these three compounds under the concentration conditions were repeatedly analyzed for five days for inter-day evaluation, and five consecutive analyses were conducted for intra-day evaluation in order to measure precision and accuracy. Thus, the reproducibility of the analysis method was verified.

### 2.4. Cell Culture and Cell Viability

Human hepatoma HepG2 cells were obtained from American Type Culture Collection and grown in Dulbecco’s Modified Eagle Medium (DMEM) (Gibco by Life Technologies, Grand Island, NE, USA) supplemented with 10% fetal bovine serum (FBS) (Gibco by Life Technologies, Grand Island, NE, USA), 100 μg/mL penicillin, and 100 μg/mL streptomycin (Hyclone, GE Healthcare Life Sciences, Logan, UT, USA). The cells were cultured at 37 °C in a humidified atmosphere of 95% air to 5% CO_2_.

### 2.5. Animal Care and Diet Preparation

Male C57BL/6 mice (7-week-old) were obtained from Samtako Korea (SAMTAKO, Inc., Osan, Korea) and used for a total of 11 weeks. Mice were given free access to water and kept at constant room temperature (22.26 °C) under 12 h dark/12 h light cycles. The mice were adapted to the new environment and food for 1 week before starting the experiment. The mice were randomly divided into the following five groups with five mice per group; Control, normal diet (*n* = 5); MCD, MCD diet (*n* = 5); DMC, demethoxycurcumin (100 mg/kg/day, *n* = 5) with MCD diet; CM, curcumin (100 mg/kg/day, *n* = 5) with MCD diet; and BDMC (100 mg/kg/day, *n* = 5) with MCD diet [45]. All 5 diet formulations were administered for 4 weeks. All mice were fasted for 1 day before sacrifice. On the day of sacrifice, a laparotomy was performed under ketamine and xylazine anesthesia (intramuscular injection of 100 mg/kg body weight and 5 mg/kg body weight, respectively), and whole-blood samples were collected via cardiac puncture. To obtain serum for biochemical determination, blood samples were centrifuged at 1000× *g* for 10 min. Livers were weighed and cut into several slices: 1 slice was fixed in 10% formalin for histological analysis; the other slices were snap-frozen in liquid nitrogen for other experiments. Serum and liver samples were then stored at −70 °C until required. All animal studies conformed to the Guide for the Care and Use of Laboratory Animals published by the US National Institute of Health (NIH Publication No. 85–23, revised 1996) based on the 3R principle (Replacement, Reduction, and Refinement) and were approved by the Institutional Animal Care and Utilization Committee for Medical Science of Wonkwang University (Approval no.WKU-15-100). Experiments were repeated twice and similar results were obtained, with the representative results being shown.

### 2.6. Cell Viability

Cell viability was examined by MTS (Promega, Madison, WI, USA) assay. Briefly, HepG2 cells were seeded at a density of 1 × 10^5^ cells/mL in 96-well plates (Nunc, A/S, Roskilde, Denmark). To determine the non-toxic concentration for cells, OA (500 μM) and DMC, CM (Sigma-Aldrich, St. Louis, MO, USA), and BDMC (TCI America, Portland, OR, USA) (10, 25, 50, 100, and 200 μM) were added to each well. The plates were then incubated for 18 h at 37 °C under 5% CO_2_. The existing medium was replaced with new DMEM supplemented with CellTiter 96^®^ AQueous One Solution Reagent (MTS solution) (5 mg/mL). After 30 min, the absorbance values were measured using an Epoch microplate spectrophotometer (BioTek, Winooski, VT, USA) at an optical density (OD) of 490 nm.

### 2.7. Oil Red O Stain in HepG2 Cells

HepG2 cells were seeded at a density of 4 × 10^5^ cells/mL in 24-well plates. To examine the fat accumulation in HepG2 cells, the cells induced by OA (500 μM) were treated for 24 h with DMC (10, 25, 50 μM), CM (25, 50, 100 μM), and BDMC (25, 50, 100 μM). The cells were rinsed with cold DPBS and fixed in 10% formalin (paraformaldehyde [Junsei Chemical Co., Ltd., Tokyo, Japan] and phosphate-buffered saline [PBS, pH 7.4]) for 1 h. After washing with 60% isopropanol, the cells were stained for at least 1 h in a freshly diluted Oil Red O solution (Sigma-Aldrich, St. Louis, MO, USA) (six parts Oil Red O stock solution and four parts H_2_O; Oil Red O Stock solution is 0.5% Oil Red O in isopropanol). Images of Oil Red O-stained cells were observed using optical microscopy (Carl Zeiss MicroImaging GmbH, Göttingen, Germany) after washing the cells with 60% isopropanol and removing the remaining Oil Red O solution. For quantitative analysis, the stained lipid droplets were dissolved with isopropanol and their absorbance was measured at 490 nm wavelengths.

### 2.8. Measurement of Lipid Levels in HepG2 Cells

HepG2 cells were seeded at a density of 4 × 10^5^ cells/mL in 24-well plates. Cells induced by OA (500 μM) were treated for 24 h with DMC (25, 50 μM), CM (50, 100 μM), and BDMC (50, 100 μM). Levels of triglycerides (TGs) and total cholesterol (TC) in HepG2 cells were quantified according to the kit protocol (BioVision, Mountain View, CA, USA).

### 2.9. Western Blot Analysis for In Vitro and In Vivo Studies

In the in vitro study, the HepG2 cells were cultured in 6-well plates (5 × 10^5^ cells/mL) and pretreated with various concentrations of DMC (25, 50 μM), CM (50, 100 μM), and BDMC (50, 100 μM). One hour later, the cells were induced with OA (500 μM) and then incubated at 37 °C. After 24 h incubation, the cell pellets were lysed with lysis buffer on ice for 20 min, and the cell debris was removed by centrifugation (16,609× *g*, 10 min, 4 °C) to obtain the cell protein sample. In terms of the in vivo analysis, liver tissue collected from sacrificed animals were homogenized in radioimmunoprecipitation assay (RIPA) lysis buffer (iNtRON Biotechnology, Daejon, Korea) on ice. The homogenates were centrifuged (16,609× *g*, 10 min, 4 °C) to obtain the upper liver tissue protein sample. The protein concentrations of cell and liver tissue samples were determined using the Bio-Rad protein assay reagent (Bio-Rad Laboratories, Hercules, CA, USA) according to the manufacturer’s instructions. The quantified samples were subjected to sodium dodecyl sulphate polyacrylamide gel (SDS-PAGE) electrophoresis and then transferred onto polyvinylidene difluoride (PVDF; Millipore, Bedford, MA, USA) membranes at 15 V for 45 min. The membranes were blocked with 3% BSA (Sigma-Aldrich, St. Louis, MO, USA) in Tris-buffered saline with Tween 20 buffer (TBST) for 2 h and were incubated with primary antibodies against ß-actin, SREBP-1c, FAS, PPAR-γ, C/EBP-α (Santa Cruz Biotechnology, Paso Robles, CA, USA), p-AMPK, AMPK, TLR-2, and TLR-4 (Cell Signaling Technology, Danvers, MA, USA) overnight at 4 °C. The membranes were then washed with TBST and incubated with anti-mouse or anti-rabbit secondary antibodies (Invitrogen, Thermo Fisher Scientific, Inc., Carlsbad, CA, USA) at room temperature. Washed membranes were then supplemented with Atto ECL plus (Tokyo, Japan), and the Image Quant LAS 4000 Mini Bio molecular Imager (GE Healthcare, Little Chalfont, Buckinghamshire, UK) was used for evaluating bands.

### 2.10. Quantitative Real-Time PCR Analysis for the In Vitro and In Vivo Studies

Total-RNA was extracted from adipocyte culture medium and mice livers using E.Z.N.A.^®^ Total RNA Kit (Omega Bio-tek, Inc.; Norcross, GA, USA) following the kit protocol. A spectrophotometer (BioTek, Winooski, VT, USA) was used to evaluate RNA purity by measuring the absorbance values at 260 and 280 nm. Reverse transcription was performed using a QuantiTect^®^ Reverse Transcription Kit (Qiagen, Hilden, Germany) and quantitative Real-Time RT-PCR was performed in triplicate using a Power SYBR^®^ Green PCR Master Mix (Applied Biosystems; Thermo Fisher Scientific, Inc. Foster City, CA, USA) with the StepOnePlus real-time RT-PCR system (Applied Biosystems; Thermo Fisher Scientific, Inc. Foster City, CA, USA). The relative gene expression levels were detected using the StepOne software v2.3 (Applied Biosystems; Thermo Fisher Scientific, Inc. Foster City, CA, USA). The sequences of the primers are listed in Table 1. The GAPDH gene was used as a loading control because it is stably and constitutively expressed at high levels in tissues and cells.

### 2.11. Histological Examination of MCD-Diet-Induced Mice

Harvested livers were fixed in 10% formalin overnight and embedded in paraffin using a tissue processor (Shandon Citadal 1000, Shandon, UK). Embedded tissues were sliced into 4-μm-thick sections using a Rotary Microtome (Microm HM340E, ThermoFisher Scientific, Waltham, MA, USA) for staining with hematoxylin and eosin (H&E). The images were obtained using the Axio Obserber Z1 microscope (Carl Zeiss MicroImaging GmbH, Göttingen, Germany).

### 2.12. Biochemical Analysis of MCD-Diet-Induced Mice

Serum TG, TC, glutamic oxaloacetic transaminase (GOT), and glutamic-pyruvic transaminase (GPT) were estimated using the enzymatic method by a commercial kit (Asan, Seoul, Korea). Liver tissues were homogenized in 0.5 mL of 1 M NaCl. Liver tissue homogenates were extracted with 3 mL of chloroform/methanol (2:1) plus 0.5 mL of 1 M NaCl. The organic phases were collected, dried, and resuspended in 0.5 M Triton X-100/methanol (2:1).

### 2.13. Cytokine Release Analysis of MCD-Diet-Induced Mice

Blood samples were collected to determine the quantity of IL-6 and TNF-α. Peripheral serum was subject to an enzyme-linked immunosorbent assay (ELISA) using an IL-6 and TNF-α kit from BD Biosciences (San Jose, CA, USA). The absorbance was read at 450 nm using a microplate reader (BioTec, Winooski, VT, USA).

### 2.14. Statistical Analysis

All results are presented as means ± standard deviation of three independent experiments. Statistical analysis was performed with IBM SPSS Statistics 24 using a one-way analysis of variance (ANOVA), which can be used to compare the means of two or more groups. A significant result means that the two means are unequal. Duncan’s Multiple Range Test (DMRT) was applied for statistically significant differences between the mean values (*p* < 0.05).

## 3. Results

### 3.1. Specificity and Linearity

**Figure 1:**
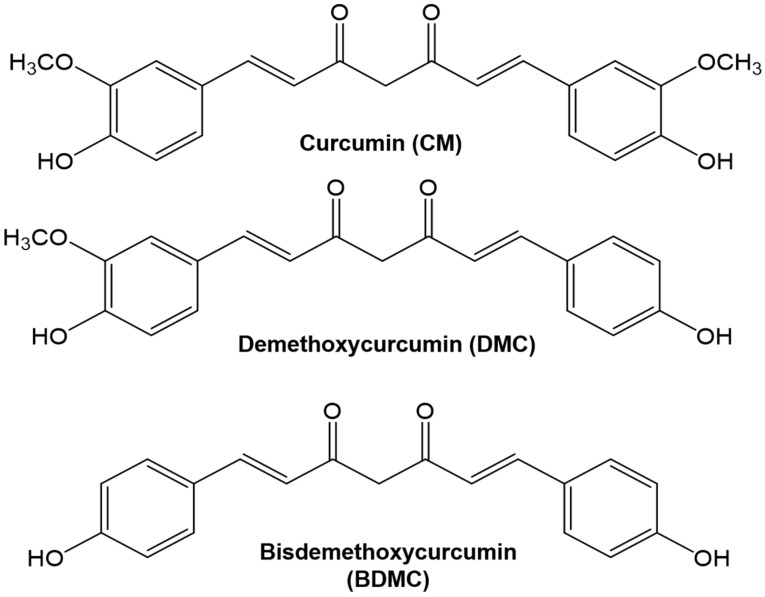
Chemical structure of curcumin, demethoxycurcumin, and bisdemethoxycurcumin.

**Figure 2:**
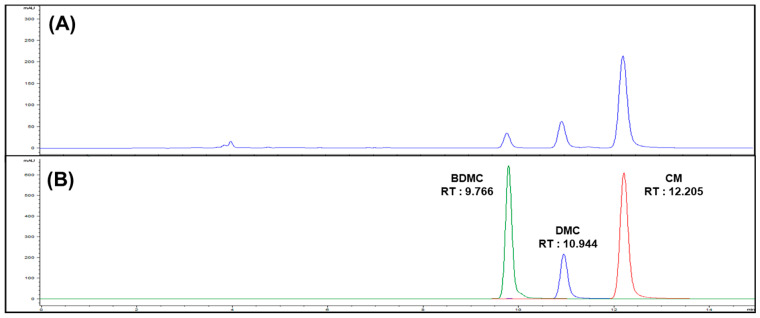
(**A**) HPLC chromatogram of turmeric extracts (Lot No. NIHHS-18-42458), (**B**) overlay chromatogram of curcuminoids.

Curcuminoids are composed of CM, DMC, and BDMC (Figure 1). HPLC equipped with a UV detector was used for analysis. The optimal solvent elution conditions were established and the retention time of the analyzed results was compared. CM was detected at 12.205 min, DMC at 10.944 min, and BDMC at 9.766 min (Figure 2). Results of the content analysis showed that CM was measured at 5.90 mg/g, BDMC at 0.94 mg/g, and DMC at 1.96 mg/g in 50% ethanolic extracts (Lot No. NIHHS-18-42458). The linear regression equation of the calibration curve for the correlation coefficient was calculated. Linearity was determined to be 0.39–100 ng/μL for CM and DMC, and 0.78–100 ng/µL for BDMC. Correlation coefficient values, which showed high degrees of linearity, were found to be 0.9999 for CM, 0.9999 for DMC, and 0.9997 for BDMC. The limit of detection (LOD) was calculated when the signal-to-noise (S/N) ratio was 3. The limit of quantitation (LOQ) was calculated when the S/N ratio was 10. The values of the method validation for curcuminoids are shown in Table 2.

### 3.2. Precision and Accuracy

To verify the method validation, HPLC analysis was conducted by setting an optimal concentration range. A spiked concentration of each sample was prepared to be 5, 10, and 20 ng/μL for CM and DMC, and 10, 20, and 25 ng/μL for BDMC. After setting the concentration of CM, DMC, and BDMC, the analysis was performed five times per day (intra-day), and the analysis was repeated for five days (inter-day) to confirm the precision and accuracy. The values of the analytical method validation are shown in Table 3.

### 3.3. Cytotoxicity of Oleic Acid and Curcuminoids in HepG2 Cells

**Figure 3:**
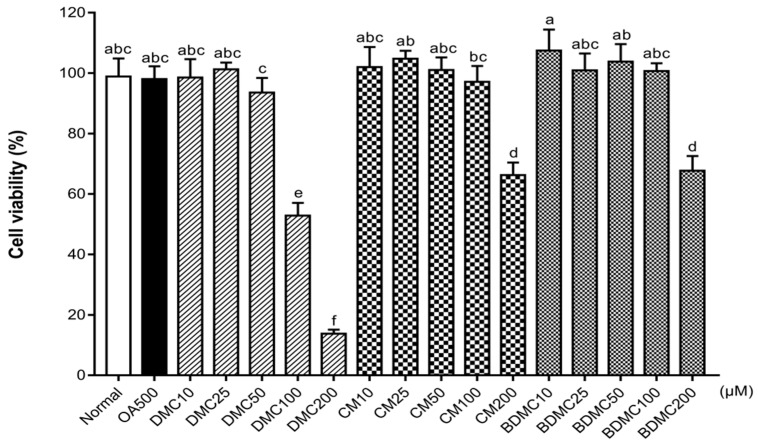
Effects of curcuminoids on cell viability in HepG2 cells. Cell viability was measured in HepG2 cells after treatment with oleic acid (OA) (500 μM), demethoxycurcumin (DMC), curcumin (CM), and bisdemethoxycurcumin (BDMC) (10, 25, 50, 100, and 200 μM) using a (3-(4,5-dimethylthiazol-2-yl)-5-(3-carboxymethoxyphenyl)-2-(4-sulfophenyl)-2H-tetrazolium) (MTS) assay. The data are means ± SD of triplicate determinations. Different letters indicate significant differences (a, c, d, e, f, ab, bc, abc) (*p* < 0.05) following Duncan’s multiple range test. OA: oleic acid; DMC: demethoxycurcumin; CM: curcumin; BDMC: demethoxycurcumin.

To evaluate the effects of oleic acid (OA), as well as DMC, CM, and BDMC, on the cell viability of human HepG2 cells, a MTS assay was conducted. When the concentration exceeds 1 mM, OA reduces cell viability and induces cell apoptosis (data not shown). In the present study, the effect of OA on lipid accumulation in hepatocarcinoma cells was measured at a concentration of 500 μM. Figure 3 illustrates that concentrations of 10 μM (DMC, CM, and BDMC), 25 μM (DMC, CM, and BDMC), 50 μM (DMC, CM, and BDMC), and 100 μM (CM and BDMC) are not cytotoxic to HepG2 cells.

### 3.4. Curcuminoids Decreased the Intracellular Lipid Accumulation in HepG2 Cells

**Figure 4:**
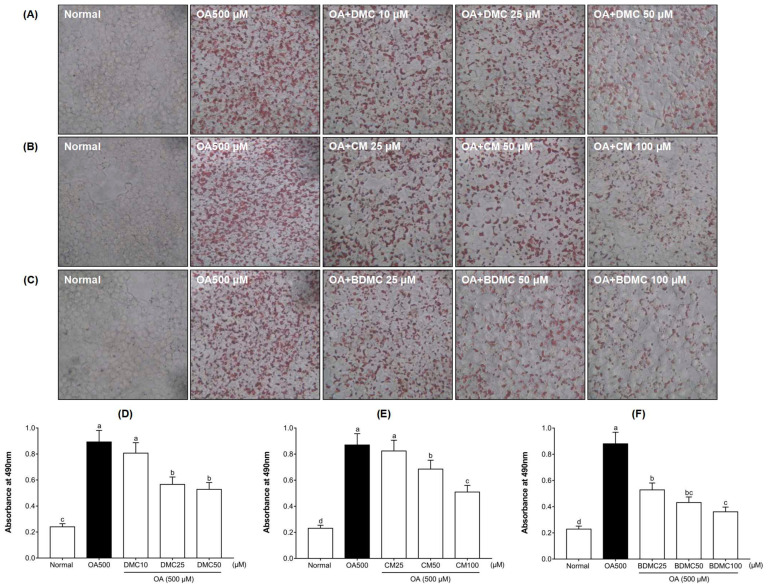
Effects of curcuminoids on intracellular lipid accumulation in HepG2 cells. Oil Red O staining of OA (500 μM)-induced HepG2 cells treated with (**A**) DMC (10, 25, and 50 μM), (**B**) CM (25, 50, and 100 μM), and (**C**) BDMC (25, 50, and 100 μM). (**D**–**F**) represent the corresponding quantitative analysis by measuring its absorbance at 490 nm. The cells were stained with Oil Red O and observed by microscope (×200). The data are means ± SD of triplicate determinations. Different letters indicate significant differences (a, b, c, d, bc) (*p* < 0.05) following Duncan’s multiple range test. OA: oleic acid; DMC: demethoxycurcumin; CM: curcumin; BDMC: demethoxycurcumin.

To verify the inhibitory effect of DMC, CM, and BDMC on OA-induced intracellular lipid accumulation, HepG2 cells were stained with Oil Red O. As shown in Figure 4A–C, after incubation with oleic acid (OA) for 24 h, the cells had significant lipid accumulation; conversely, no lipid droplets were found in the untreated cells. However, the presence of curcuminoids weakened OA-mediated Oil Red O stains in a dose-dependent manner. As shown in Figure 4D–F, the quantification of the extracted Oil Red O dye revealed that lipid accumulation in HepG2 cells increased by 4-fold with the addition of OA. When cells were incubated with OA plus curcuminoids, cellular lipid inclusions were reduced by 1.1–2.4-fold compared with the cells treated with OA alone. In addition, the effect of BDMC on inhibiting lipid accumulation was more noticeable than that of DMC or CM.

### 3.5. Curcuminoids Decreased Triglyceride (TG) and Total Cholesterol (TC) Levels in HepG2 Cells

**Figure 5:**
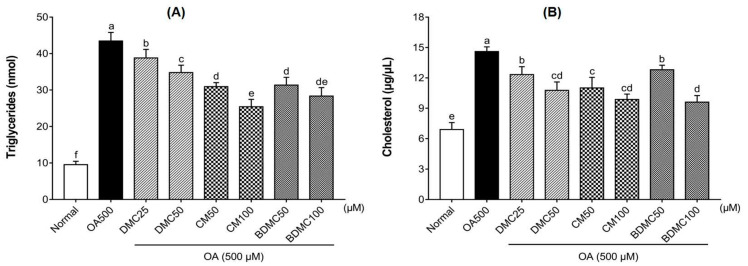
Effects of curcuminoids on triglyceride (TG) and total cholesterol (TC) levels in HepG2 cells. The enzymatic colorimetric method was used to analyze the total intracellular triglyceride (**A**) and total cholesterol (**B**) produced by OA (500 μM)-induced HepG2 cells treated with DMC (25 and 50 μM), CM (50 and 100 μM), and BDMC (50 and 100 μM). The data are means ± SD of triplicate determinations. Different letters indicate significant differences (a, b, c, d, e, f, cd, de) (*p* < 0.05) following Duncan’s multiple range test. OA: oleic acid; DMC: demethoxycurcumin; CM: curcumin; BDMC: demethoxycurcumin.

To examine the effect of DMC, CM, and BDMC on biochemical changes, we determined the level of TGs and TC in OA-induced HepG2 cells. Compared with the untreated normal group, the TG and TC levels were significantly elevated under induction with OA. When treated with curcuminoids, a downward trend could be observed compared with the negative control group. Additionally, a high concentration of curcuminoids showed more pronounced inhibitory effects (Figure 5).

### 3.6. Curcuminoids Inhibited the Gene and Protein Expression of Hepatic Lipogenic Markers in HepG2 Cells

**Figure 6:**
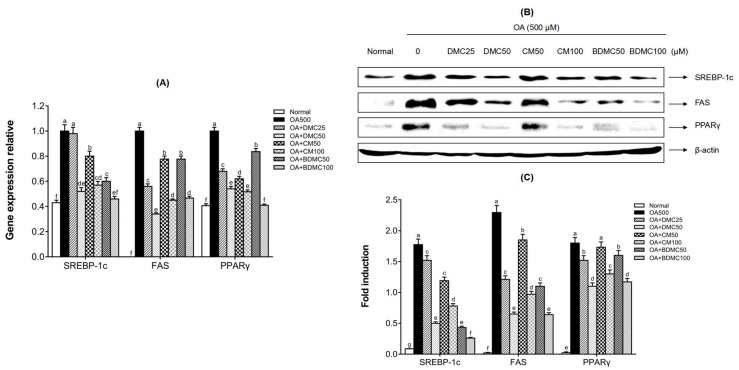
Effects of curcuminoids on hepatocyte lipid accumulation at both the protein and mRNA expression levels. (**A**) The gene expression of SREBP-1c, FAS, and PPARγ in HepG2 cells was detected by real time RT-PCR, in which glyceraldehyde-3-phosphate dehydrogenase (GAPDH) served as an internal control. (**B**) The protein expressions of SREBP-1c, FAS, and PPARγ in HepG2 cells were detected by Western blot analysis, in which β-actin served as an internal control. (**C**) The quantification of the immunoblot by densitometry. The data are means ± SD of triplicate determinations. Different letters in the same group indicate significant differences (a, b, c, d, e, f, g, cd, de, ef) (*p* < 0.05) following Duncan’s multiple range test. OA: oleic acid; DMC: demethoxycurcumin; CM: curcumin; BDMC: demethoxycurcumin.

Real time RT-PCR and Western blot analysis were performed to determine the mechanism of curcuminoids to suppress hepatic lipid accumulation in protein and mRNA levels. Figure 6 reveals that the gene and protein expression of adipogenis transcription factors and enzymes significantly increased in the presence of OA. However, according to Figure 6A, the treatment of DMC, CM, and BDMC significantly hindered the increase in gene expression of SREBP-1, FAS, and PPARγ. In addition, as shown in Figure 6B,C, the expression of these three proteins related to lipid accumulation was reduced in the presence of DMC, CM, and BDMC. All curcuminoids appeared to inhibit lipid accumulation in a dose-dependent manner. 

### 3.7. Curcuminoids Increased the AMPK Phosphorylation Level in HepG2 Cells

**Figure 7:**
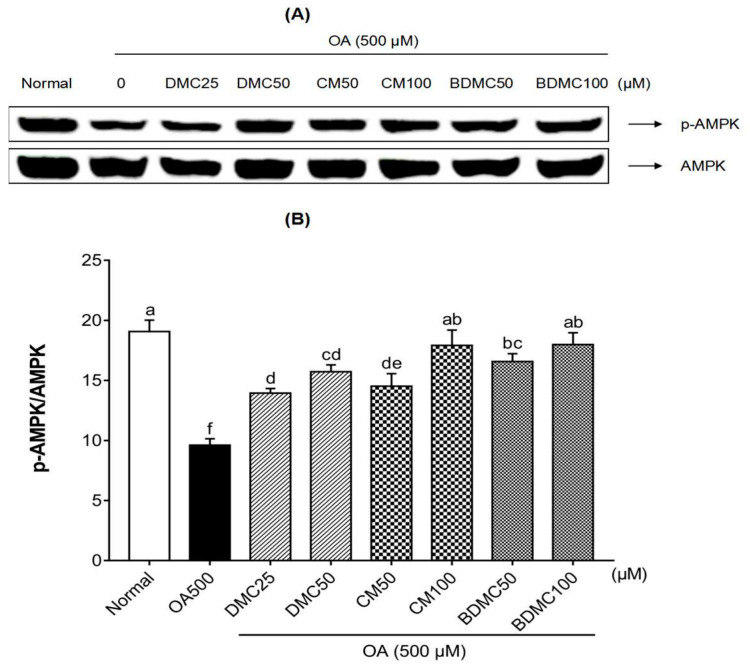
Effects of curcuminoids on adenosine monophosphate-activated protein kinase (AMPK) phosphorylation in HepG2 cells. (**A**) Phosphorylated AMPK in HepG2 cells was detected by Western blot analysis. Expression levels were normalized to that of the total AMPK protein. (**B**) The quantification of the immunoblot by densitometry. The data are means ± SD of triplicate determinations. Different letters indicate significant differences (a, d, f, ab, bc, cd, de) (*p* < 0.05) following Duncan’s multiple range test. OA: oleic acid; DMC: demethoxycurcumin; CM: curcumin; BDMC: demethoxycurcumin.

Activated AMPK can inhibit lipid accumulation in adipocytes, and is considered to be a key functional enzyme for managing liver steatosis. To investigate the mechanism of curcuminoids on the AMPK pathway, we examined the effect of DMC, CM, and BDMC treatment on AMPK phosphorylation in OA-induced HepG2 cells by Western blot analysis. As shown in Figure 7B, OA treatment reduced phosphorylated AMPK by 2 times compared to the normal group. However, the image of the Western blot bands showed that the decrease in AMPK phosphorylation was reversed by treatment of DMC, CM, and BDMC (Figure 7A). According to the quantitative results of Western blotting, the treatment of these three curcumins increased the phosphorylation of AMPK by 1.5–1.9 times (Figure 7B). Among them, the treatment of 100 μ CM and 100 μM BDMC made the activation of AMPK reach the normal group level. Additionally, the expression of phosphorylated AMPK was increased in a dose-dependent manner.

### 3.8. Curcuminoids Improved the Abnormalities of Body and Liver Weight in Mice Fed with an MCD Diet

**Figure 8:**
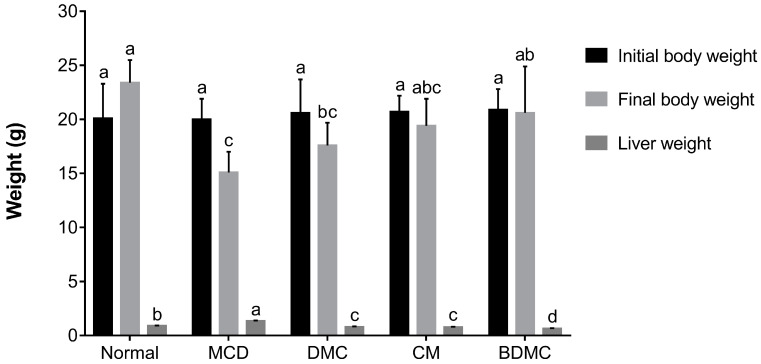
Effect of curcuminoids on methionine choline deficient (MCD)-diet body weight and liver weight. Data are expressed as mean ± SD of 7 animals. Different letters in the same line represent significant differences (a, b, c, d, ab, bc, abc) (*p* < 0.05) following Duncan’s multiple range test. DMC: demethoxycurcumin; CM: curcumin; BDMC: demethoxycurcumin.

The initial weight and final weight of the mice are summarized in Figure 8. Compared with mice on a normal diet, the mean body weight of mice fed the MCD diet for four weeks was significantly reduced. When curcuminoids were administered orally, the weight loss of mice caused by the MCD diet was significantly relieved; in particular, when treated with BDMC, the alleviation effect was strengthened. Regarding the liver weight, when compared with the normal group, the MCD-diet group increased significantly, representing hepatic damage. Liver weight changes that occurred when curcuminoids were taken orally showed a high correlation with the changes in body weight. The results indicated a certain hepatoprotective effect of curcuminoids and BDMC showed the most obvious effect.

### 3.9. Curcuminoids Decreased the Blood Index of Liver Injury Induced by the MCD Diet

**Figure 9:**
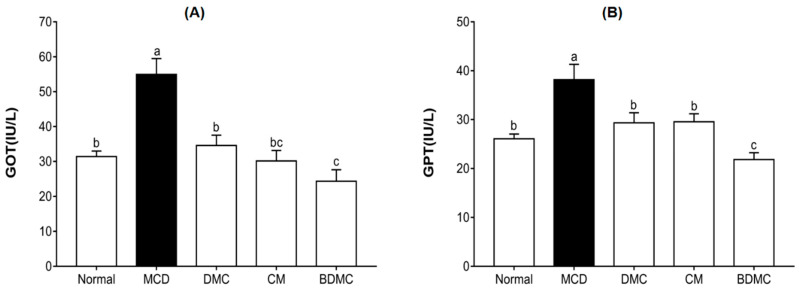
Effects of curcuminoids on glutamic oxaloacetic transaminase (GOT) and glutamic-pyruvic transaminase (GPT) levels in mice. Blood samples were collected from C57BL/6J mice fed a normal diet, the methionine choline deficient (MCD) diet, or the same MCD diet supplemented with curcuminoid treatment for four weeks, and plasma GOT (**A**) and GPT (**B**) levels were determined. The data are means ± SD of triplicate determinations. Different letters indicate significant differences (a, b, c, bc) (*p* < 0.05) following Duncan’s multiple range test. DMC: demethoxycurcumin; CM: curcumin; BDMC: demethoxycurcumin.

When liver cells are necrotic, a large amount of glutamic oxaloacetic transaminase (GOT) and glutamic-pyruvic transaminase (GPT) will be released into the blood; thus, it is an important indicator to judge liver damage [50]. As shown in Figure 9A, compared with the normal group, the GOT level of the MCD-diet group increased by 1.8 times. Compared with the MCD-diet negative control group, the GOT levels decreased by 1.6–2.3-fold under the treatment of the three curcuminoids. Similarly, Figure 9B indicated that the MCD diet increased the GPT level by 1.5 times compared to the normal group. However, the DMC, CM, and BDMC treatments decreased the GPT levels by 1.3–1.8-fold compared with the MCD-diet negative control group. Based on the results of elevated plasma GOT and GPT levels in mice fed the MCD diet for four weeks, we speculated that this group of mice developed severe steatosis. In addition, BDMC showed a more significant effect on reducing GOT and GPT levels than DMC and CM, which further supports the hypothesis that BDMC shows a stronger effect of inhibiting lipogenesis among the three curcuminoids.

### 3.10. Curcuminoids Inhibited the Hepatic Steatosis Induced by the MCD Diet

**Figure 10:**
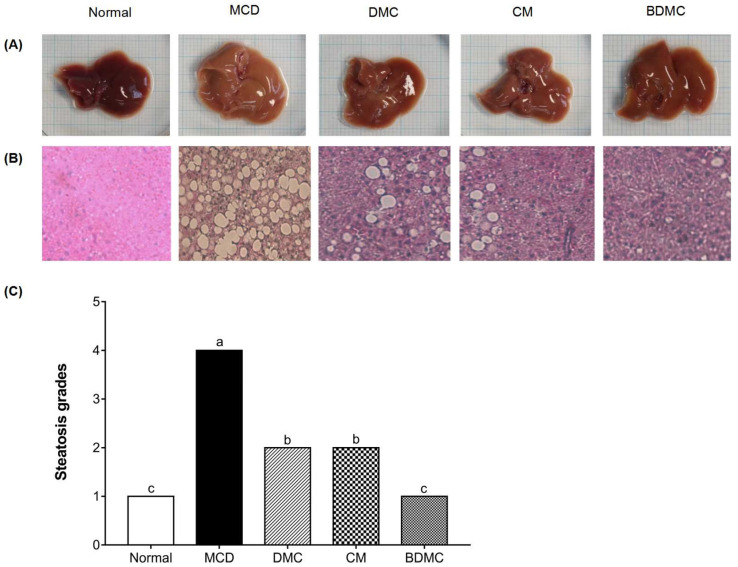
Histological analysis of liver steatosis and liver morphology. (**A**) Photographs of mouse livers. (**B**) Hematoxylin and eosin staining of liver sections obtained from C57BL/6J mice fed a normal diet, the methionine choline deficient (MCD) diet, or the same MCD diet supplemented with curcuminoid treatment for four weeks, original magnification, ×400. (**C**) The grades of steatosis, Grade 1 (<5%), Grade 2 (5–33%), Grade 3 (>33–66%), and Grade 4 (>66%). The data are means ± SD of triplicate determinations. Different letters indicate significant differences (a, b, c) (*p* < 0.05) following Duncan’s multiple range test. DMC: demethoxycurcumin; CM: curcumin; BDMC: demethoxycurcumin.

To investigate the effects of curcuminoids on MCD-diet-induced liver tissue injury, liver histopathologic changes were examined. Compared with the normal-diet group, the MCD-diet group indicating lipid accumulation (yellow liver necrosis). However, it was observed that the steatosis was relatively suppressed in curcuminoid-treated groups (Figure 10A). In addition, we further studied the hepatic pathological changes and H&E staining was used to visualize liver lipid content. Morbid histology was observed in the MCD-diet samples, whereas curcuminoid administration hampered the lipid droplet accumulation (Figure 10B). In particular, the mice treated with BDMC showed more normal morphology and histology, indicating that it is more likely to be a hepatoprotective agent than DMC and CM (Figure 10C).

### 3.11. Curcuminoids Decreased Lipid Accumulation Induced by the MCD Diet

Levels of serum TC, TG, low-density lipoprotein (LDL), and high-density lipoprotein (HDL) were detected to evaluate the effect of curcuminoids on biochemical changes. The TC, TG, and LDL levels significantly increased in the MCD-diet group compared to those in the normal-diet group. In addition, compared with the MCD-negative control group, the lipid accumulation was relatively decreased in the curcuminoid-treated groups. DMC and CM showed similar effectiveness, whereas the BDMC group still represented a more obvious effect (Table 4).

### 3.12. Curcuminoids Inhibited the Protein and Gene Expression of Hepatic Lipogenic Markers In Vivo

**Figure 11:**
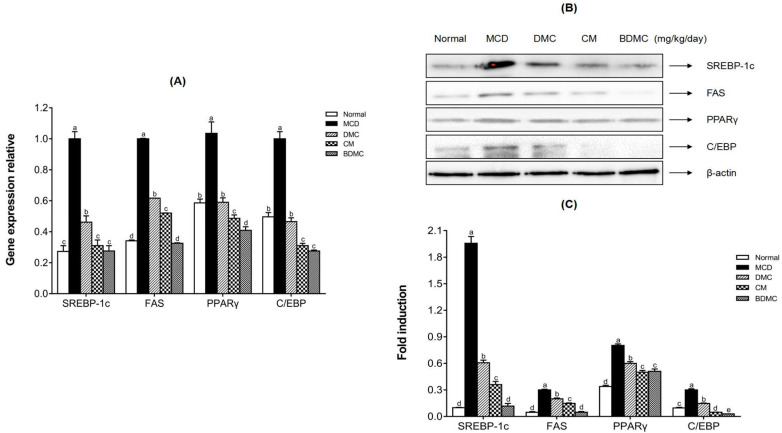
Effects of curcuminoids on the protein and mRNA expression levels of hepatic lipid accumulation markers. (**A**) The mRNA expression levels of SREBP-1c, FAS, PPARγ, and C/EBP in liver tissues were evaluated by real-time RT-PCR and normalized to GAPDH levels. (**B**) The proteins related to lipogenesis in liver tissues were detected by Western blot analysis. Expression levels were normalized to those of the β-actin protein. (**C**) The quantification of the immunoblot by densitometry. The data are means ± SD of triplicate determinations. Different letters in the same group indicate significant differences (a, b, c, d, e) (*p* < 0.05) following Duncan’s multiple range test. MCD: methionine choline deficient; DMC: demethoxycurcumin; CM: curcumin; BDMC: demethoxycurcumin.

To understand the effect of curcuminoids on the protein and gene expression of adipogenic transcription factors and enzymes in the fatty liver model induced by the MCD diet, qRT-PCR and Western blot analysis were used to observe the changes of SREBP-1c, FAS, PPARγ, and C/EBP levels in the liver tissues of experimental animals. From the results of Figure 11A, the gene expression of SREBP-1c, FAS, PPARγ, and C/EBP, significantly increased in the MCD-diet group, but they were suppressed after treatment with curcuminoids. In addition, the protein expression of SREBP-1c, FAS, PPARγ, and C/EBP also increased in the MCD-diet group, and decreased after drug treatment, which was consistent with the results of the gene expression analysis (Figure 11B,C). In particular, the expressions of SREBP and FAS in the BDMC-treated group decreased almost to the same levels as those of the normal-diet group, both in terms of protein and gene levels. In conclusion, the results showed that CM and BDMC had stronger inhibitory effects than DMC, at both the protein and the mRNA level.

### 3.13. Curcuminoids Increased the AMPK Phosphorylation Level In Vivo

**Figure 12:**
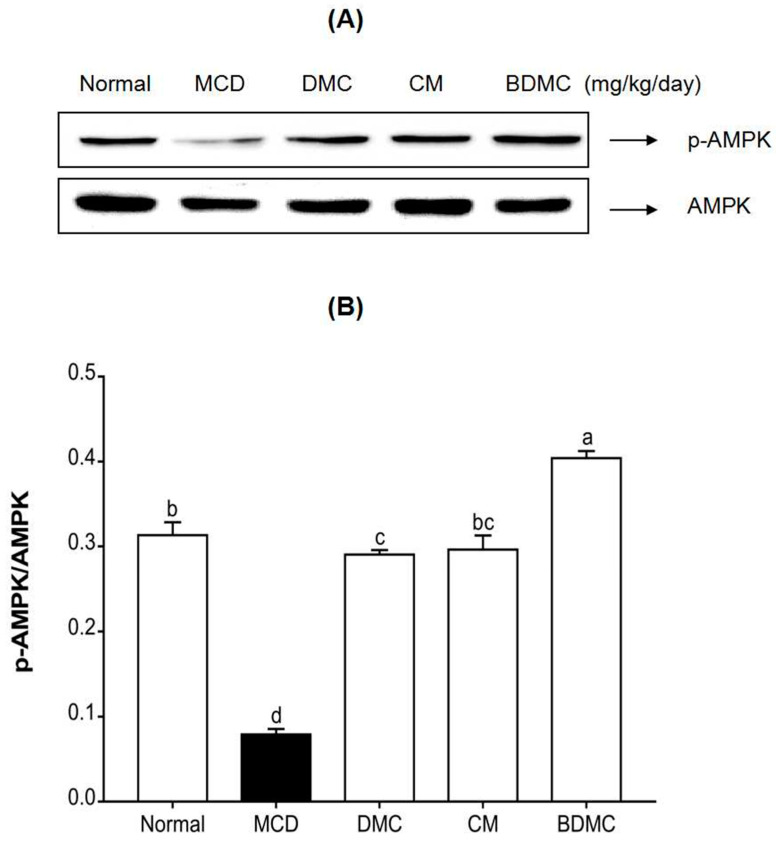
Effects of curcuminoids on 5’ AMP-activated protein kinase (AMPK) phosphorylation in mice. (**A**) AMPK phosphorylation in liver tissues was detected by Western blot analysis. Expression levels were normalized to that of the AMPK protein. (**B**) The quantification of the immunoblot by densitometry. The data are means ± SD of triplicate determinations. Different letters indicate significant differences (a, b, c, d, bc) (*p* < 0.05) following Duncan’s multiple range test. MCD: methionine choline deficient; DMC: demethoxycurcumin; CM: curcumin; BDMC: demethoxycurcumin.

Activated AMPK has the ability to inhibit the synthesis of fatty acids and cholesterol, so it is considered as a potential target for NAFLD treatment. To investigate the effect of curcuminoids on AMPK activation of liver proteins, the phosphorylation of AMPK was confirmed by Western blot analysis. According to the bands in Figure 12A, it can be seen intuitively that the phosphorylation of AMPK in the MCD-diet group was significantly lower than that in the normal-diet group, and the treatment of the three curcuminoids reversed this inhibition. According to the quantitative results shown in Figure 12B, compared with the normal-diet group, the phosphorylation of AMPK in the MCD-diet group decreased 4-fold. However, the curcuminoids treatment increased phosphorylation level of AMPK by 3.7–5.1-fold compared with the MCD-diet group. Additionally, a more noticeable increase was observed in the BDMC-treated group compared to the DMC- and CM-treated groups.

### 3.14. The Anti-Inflammatory Effects of Curcuminoids

**Figure 13:**
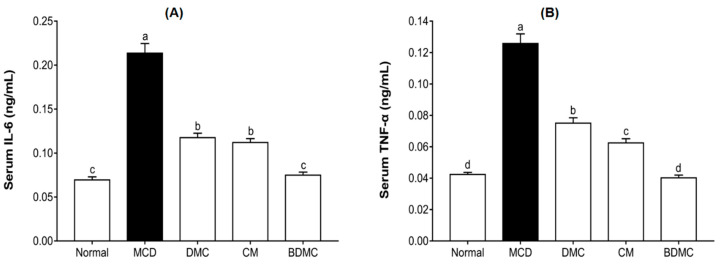
Effects of curcuminoids on interleukin (IL)-6 and tumor necrosis factor (TNF)-α concentrations in mice. Blood samples were collected, and plasma IL-6 (**A**) and TNF-α (**B**) concentrations were determined. The data are means ± SD of triplicate determinations. Different letters indicate significant differences (a, b, c, d) (*p* < 0.05) following Duncan’s multiple range test. MCD: methionine choline deficient; DMC: demethoxycurcumin; CM: curcumin; BDMC: demethoxycurcumin.

**Figure 14:**
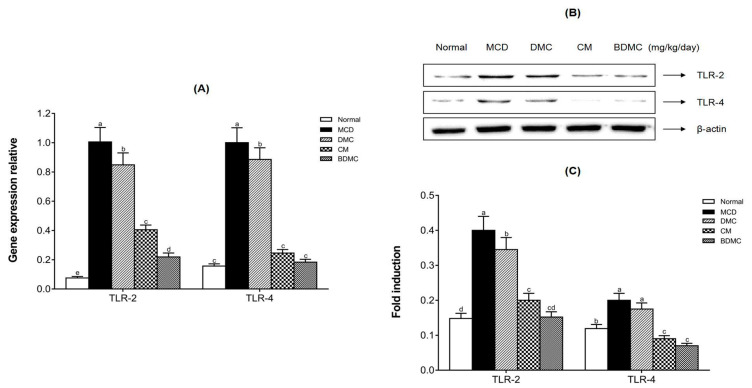
Effects of curcuminoids on toll-like receptor protein and mRNA expression in mice. (**A**) The mRNA expression levels of TLR-2 and TLR-4 in liver tissues were detected by real-time RT-PCR, and GAPDH served as an internal control. (**B**) Toll-like receptor-2 (TLR-2) and toll-like receptor-4 (TLR-4) protein expression levels in liver tissues were detected by Western blot analysis. Expression levels were normalized to those of the β-actin protein. (**C**) The quantification of the immunoblot by densitometry. The data are means ± SD of triplicate determinations. Different letters in the same group indicate significant differences (a, b, c, d, e, cd) (*p* < 0.05) following Duncan’s multiple range test. MCD: methionine choline deficient; DMC: demethoxycurcumin; CM: curcumin; BDMC: demethoxycurcumin.

As shown in Figure 13A, compared with the normal-diet group, the serum IL-6 level of the MCD-diet group increased 3.1 times. The curcuminoids treatment reduced IL-6 levels by 1.8–2.9-fold. Similarly, as shown in Figure 13B, the serum TNF-α level of the MCD-diet group was 3 times that of the normal-diet group. DMC, CM, and BDMC treatment suppressed the increase in TNF-α levels caused by the MCD diet, reducing it by 1.7–3.1-fold. In addition, as upstream receptors that trigger these cytokines, TRL-2 and TRL-4 were also assessed by measuring mRNA and protein levels. Figure 14 shows that the mRNA and protein expression of TRL-2 and TRL-4 increased in the MCD-diet group and decreased in the curcuminoid groups. According to the gene expression shown in Figure 14A, the MCD diet increased the gene expression of TLR-2 and TLR-4 by 13.3 times and 6.4 times, respectively. The curcuminoids treatment reduced TLR-2 gene expression by 1.2–4.6 times and TLR-4 gene expression by 1.1–5.4 times. According to the Western blot results shown in Figure 14B, the increase in the expression of TLR-2 and TLR-4 caused by the MCD diet was significantly suppressed by curcuminoids. The quantitative results showed that, compared with the MCD-diet negative control group, the treatment of DMC, CM, and BDMC reduced the expression of TLR-2 and TLR-4 by 1.2–2.6-fold and 1.1–2.9-fold, respectively (Figure 14C). In particular, the levels of TRL-2 and TRL-4 in the CM and BDMC groups were significantly lower than those in the DMC group.

## 4. Discussion

Among the curcuminoids analyzed for content, CM was the highest whereas the content of BDMC was the lowest. By summarizing the results of the method validation for the curcuminoids, it was confirmed that the values came within the range of the guidelines specified by the Korea Food Safety Assessment Service.

Nonalcoholic fatty liver disease (NAFLD) has continuously increased in tandem with population age and obesity because NAFLD is closely associated with metabolic syndrome and visceral obesity [51]. Hepatic lipid accumulation produces multiple signals that can alter the metabolism of glucose and lipids, which leads to the presence of intracellular fat vacuoles, in addition to a lack of the capability to perform the mitochondrial β-oxidation processes, development of proinflammatory mechanisms, and generation of oxidative stress, ultimately leading to hepatocellular apoptosis [52]. According to the “two-hit theory” mentioned previously, excessive lipid accumulation in the liver and oxidative stress are believed to be the predominant pathogenic mechanisms of NAFLD, so treatment measures for NAFLD should be investigated in terms of hepatic lipid regulating and anti-oxidants [53].

Turmeric is prepared from the roots of the perennial herb *C. longa*, a member of the Zingiberaceae family, and has been applied in traditional medicine and as a food additive [54]. Many phenolic compounds with research value are derived from the dried rhizome of turmeric, and are known as curcuminoids. Curcumin (CM), demethoxycurcumin (DMC), and bisdemethoxycurcumin (BDMC) are three major natural polyphenol analogues extracted from turmeric [55], and our results from the HPLC analysis using turmeric extracts (Lot No. NIHHS-18-42458) also showed that CM, DMC, and BDMC were mainly included (Figure 2). Our previous study reported that the 50% ethanol extract of *C*. *longa* improved the regulation of lipid accumulation in an MCD-diet-induced NAFLD model by decreasing lipogenesis [56]. In terms of a single compound, several studies have proved the hepatoprotective effect of CM by ameliorating hepatic lipid accumulation and lipid peroxidation related to various conditions [57,58]. Investigations using DMC and BDMC have been limited because of their low content in, and difficult separation from, turmeric [59]. However, because the chemical structures of DMC and BDMC are similar with that of CM, we assumed that the physiological activities of DMC and BDMC would be similar to those of CM. Therefore, in the present study, we compared the potential inhibitory ability of the three major curcuminoids (CM, DMC, and BDMC) on lipogenesis using OA-induced HepG2 cells and MCD-diet mice models.

The influence of curcuminoids on MCD-diet-induced liver injury is directly related to changes in the body and liver weight of mice. According to our measurement results on the weight of mice (Figure 8), after four weeks of the MCD diet, compared with the normal-diet group, the body weight declined significantly, and the liver weight evidently increased. According to the excessive differences found between the body weight and liver of the MCD and control animals, we speculate that the increase in liver weight of mice may be caused by the accumulation of liver lipids due to the lack of methionine and choline, while the weight loss of mice may be caused by the accumulation of lipids in the liver, which hinders the normal function of the liver. However, the treatment with curcuminoids reversed the influence of the MCD diet on the body weight and liver weight of the mice, making the weight approach the value of the normal-diet group. In particular, when the MCD diet was supplemented with BDMC, it was found that the effects on liver protection were surprisingly stronger than using DMC and CM, which may be attributed to its methoxy substitution in the chemical structure. In addition, the MCD diet can cause a dysfunction of liver lipids and induce fatty liver. The accumulation of lipid droplets in the cytoplasm of large or small microvesicles in hepatocytes is a manifestation of hepatic steatosis. Oil Red O staining (Figure 4) and H&E staining (Figure 10) depict that the three curcuminoids have a strong inhibitory effect on steatosis caused by OA treatment and the MCD diet. In particular, the intrahepatic lipid droplets of the BDMC-treated mice almost completely disappeared, which again verified the outstanding liver-protecting effect of BDMC, and heightened the significance of this study to compare the efficacy of the three curcuminoids. To understand the effect of curcuminoids on biochemical profiles in OA-induced HepG2 cells and MCD-diet mice, the lipids contained in hepatocarcinoma cells and the serum of experimental animals were determined. The inhibitory effect of curcuminoids on TG and TC accumulation was obvious both in the in vitro (Figure 5) and in vivo (Table 4) studies. These discoveries laid the foundation for this study to further explore the inhibitory effect of DMC, CM, and BDMC on lipogenesis from a molecular perspective.

Figure 6, Figure 7, Figure 11 and Figure 12 reveal the protein and gene expression of molecules involved in lipid synthesis in the in vitro and in vivo analysis. SREBP-1c, as a major transcription factor controlling the expression of cholesterol and fatty acid metabolism-related enzyme genes, plays a highly important role in lipogenesis, insulin sensitivity, and fatty acid homeostasis [25]. In the present study, whether in the in vitro or in vivo analysis of SREBP-1c, it can be concluded that BDMC has a stronger hepatoprotective effect than CM and DMC. In addition, fatty acid synthase (FAS) is a downstream target gene regulated after SREBP activation, and is known as a lipogenic rate-limiting enzyme that mainly mediates the synthesis of fatty acids [60]. Both in the HepG2 cell and MCD-diet mice models of this study, the expression of FAS showed consistent results with SREBP-1c, which revealed that BDMC have a more obvious effect in preventing lipogenesis. The expression of CCAAT/enhancer-binding proteins (C/EBPs) and Peroxisome proliferator-activated receptors (PPARs) control the differentiation of adipocytes and induce lipogenesis [61]. In the in vitro investigation, the three curcuminoids significantly inhibited the expression of PPARγ protein level in hepatocarcinoma cells. Regarding the in vivo analysis, the results also indicated the substantial inhibitory effect of curcuminoids on adipogenesis, with the CM- and BDMC-treated groups demonstrating a more obvious effect than the DMC-treated group. Additionally, in treatment of MCD-diet mice, when using CM and BDMC, the expression of C/EBP protein and mRNA levels showed a marked downward trend compared to DMC, which may be attributed to the regulation of AMPK. Activated AMPK inhibits the synthesis of fatty acids and cholesterol in the process of ATP consumption, and promotes the oxidation of fatty acids and the corresponding process of producing ATP [62]. Emerging evidence suggests that phosphorylated AMPK inhibits the downstream target proteins C/EBP, PPARγ, and SREBP-1c through inhibiting preadipocyte differentiation, reducing liver TG levels, and thus ameliorating steatosis [63]. Figure 7 and Figure 12 display the Western blot data of AMPK phosphorylation in HepG2 cells and MCD-diet mice, respectively. The addition of OA to HepG2 cells and feeding mice with an MCD diet resulted in a significant decrease in phosphorylated AMPK, and the treatment of curcuminoids reduced lipid accumulation significantly. In the in vivo analysis, when treated with BDMC, there was a clear growth trend, higher than that of the normal-diet group. This is a somewhat remarkable result that may suggest BDMC treatment provides a strong ability to inhibit lipid synthesis and may even cause the lipid content to be lower than normal. Collectively, the findings described above indicate that the regulation of these lipid metabolism-related transcription factors support the conjecture that curcuminoids have an ameliorative effect on NAFLD, and provide favorable evidence that the effect of DMC is weaker than that of the other two curcuminoids.

Chronic systemic inflammation can lead to ectopic deposition of lipids, causing endoplasmic reticulum stress, oxidative stress, and cell apoptosis. Studies have shown that there is a close relationship between liver steatosis and chronic inflammation [64]. To investigate the anti-inflammatory property of curcuminoids, we determined the expression of common inflammatory markers, such as the toll-like receptors (TLRs) on the surface of innate immune cells, hepatitis cytokines tumor necrosis factor (TNF)-α, and interleukin (IL)-6 in MCD-diet mice. TLR is a single trans-membrane, non-catalytic protein that can recognize the pathogen association molecular pattern (PAMP) and damage association molecular pattern (DAMP) to initiate the innate immune response [65]. The stimulation of TLRs leads to the activation of NF-κB and mitogen-activated protein kinase (MAPK) signal pathways, consequently synthesizing and releasing inflammatory cytokines. Pro-inflammatory cytokines, such as TNF-α and IL-6, are the important manifestations of the progression from NAFL to NASH [66]. Figure 13 illustrates that curcuminoids possess a significant inhibitory effect on the expression of TNF-α and IL-6, and BDMC still showed the strongest inhibitory effect, even reducing cytokines to normal levels. We suppose that the abatement of inflammatory cytokines may be caused due to a reduction of TLRs. Therefore, the present study analyzed the influences of curcuminoids on TLRs to verify this possibility. The results revealed that curcuminoids have the effect of suppressing the expression of TLR-2 and TLR-4 both at the protein and mRNA levels (Figure 14). Additionally, stronger inhibitory effects were found in CM and BDMC treatment, which implies that BDMC and CM have a greater value for NAFLD treatment and prevention.

There is little existing research concerning the treatment of NAFLD with curcumin derivatives. This study is the first to report the inhibitory activity of DMC on NAFLD, and compares the three main curcuminoids of turmeric in the prevention and treatment of NAFLD, which provides a more comprehensive and in-depth understanding of curcumin’s hepatoprotective activity. However, the in vitro experiments in this study were performed only with HepG2 cells, and the reproducibility of the results of different hepatic cell lines is unknown, which is also the limitation of this study. In addition, the excellent liver-protecting activity of curcumin derivatives shown in animal experiments and cell experiments is not necessarily suitable for humans. Since clinical verification is the limitation of this study, further clinical research on curcumin derivatives is an inevitable way to strengthen the credibility of our claim that curcuminoids provide an efficient novel treatment for NAFLD.

## 5. Conclusions

In the present study, we established an analysis method of quantitation and verified the analysis for curcuminoids using HPLC equipped with a UV detector. It was confirmed that the analysis method has the desired specificity, linearity, precision, and accuracy. Furthermore, in vitro and in vivo analyses were conducted to assess the curative effect of curcuminoids on NAFLD, and compare the inhibitory effectiveness of DMC, CM, and BDMC on lipogenesis. The results comprehensively confirmed the hepatoprotective effect of the three curcuminoids on eliminating liver lipid accumulation, and clearly indicated that BDMC and CM may have a more effective therapeutic effect on NAFLD. These current findings have significant implications for the clinical application prospects of curcuminoids, and further clinical studies are needed to verify their possibility of providing a more efficient novel treatment proposal for NAFLD.

## Figures and Tables

**Table 1:** Primer sequences used for real-time RT-PCR.

Target Genes	Primer Sequences (5′-3′)	
	Forward Primer	Reverse Primer
human		
*SREBP-1c*	CGCAAGGCCATCGACTACAT	GACTTAGGTTCTCCTGCTTGAGTTTC
*FAS*	AAGGACCTGTCTAGGTTTGATGC	TGGCTTCATAGGTGACTTCCA
*PPARγ*	GCAGGCTCCACTTTGATT	ACCACTCCCACTCCTTTG
*GAPDH*	CAGGGCTGCTTTTAACTCTGGT	GATTTTGGAGGGATCTCGCT
mouse		
*SREBP-1c*	AGGCCATCGACTACATCCG	TCCATAGACACATCTGTGCCTC
*C/EBP*	GCCGAGATAAAGCCAAACAA	CCTTGACCAAGGAGCTCTCA
*PPARγ*	TGTGGGGATAAAGCATCAGGC	CCGGCAGTTAAGATCACACCTAT
*FAS*	AGAGATCCCGAGACGCTTCT	GCCTGGTAGGCATTCTGTAGT
*TLR-2*	GTACGCAGTGAGTGGTGCAAGT	GGCCGCGTCATTGTTCTC
*TLR-4*	AATCCCTGCATAGAGGTACTTCCTAAT	CTCAGATCTAGGTTCTTGGTTGAATAAG
*GAPDH*	AACTTTGGCATTGTGGAAGG	GGATGCAGGGATGATGTTCT

**Table 2:** Parameters of the calibration curve, limit of detection (LOD), and limit of quantification (LOQ) for HPLC method validation.

Compound	RT ^a^ (min)	Calibration Curve	*R* ^2 b^	Linear Range (ng/μL)	LOD ^c^ (ng/μL)	LOQ ^d^ (ng/μL)	Amount (mg/g)
CM	12.205	*y =* 98.873*x* + 2.4127	0.9999	0.39–100	1.16	3.50	5.90
DMC	10.944	*y =* 94.159*x* − 21.032	0.9999	0.39–100	1.03	3.11	1.96
BDMC	9.766	*y =* 110.33*x* − 87.724	0.9997	0.78–100	2.53	7.67	0.94

^a^ RT, retention time. ^b^ *R*^2^, coefficient of correlation. ^c^ LOD, limit of detection (signal-to-noise (S/N) = 3). ^d^ LOQ, limit of quantification (S/N = 10).

**Table 3:** Accuracy and precision of data for curcuminoids.

Compound	Spiked Conc. (ng/µL)	Intra-Day Precision (*n* = 5)			Inter-Day Precision (*n* = 5)		
		Measured ^a^ (ng/µL)	RSD ^b^ (%)	Accuracy ^c^ (%)	Measured (ng/µL)	RSD (%)	Accuracy (%)
CM	5	4.76 ± 0.03	0.69	95.15	4.82 ± 0.04	0.91	96.50
	10	9.90 ± 0.13	1.28	98.97	9.73 ± 0.04	0.43	97.30
	20	20.09 ± 0.11	0.56	100.44	20.20 ± 0.17	0.83	100.99
DMC	5	4.84 ± 0.24	4.90	96.84	4.98 ± 0.02	0.41	99.53
	10	10.64± 0.21	1.94	106.43	9.46 ± 0.07	0.70	94.64
	20	21.39 ± 0.67	3.14	106.97	19.40 ± 0.15	0.78	97.02
BDMC	10	9.49 ± 0.32	3.48	94.92	10.07 ± 0.18	1.88	100.72
	20	19.75 ± 0.54	2.89	98.76	20.09 ± 0.29	1.50	100.45
	25	23.81 ± 0.67	2.96	95.25	25.28 ± 0.51	2.09	101.12

^a^ All values are means ± SD. ^b^ RSD, relative standard deviation. ^c^ Accuracy (%) = (Measured value/spiked value) × 100.

**Table 4:** Biochemical liver function effects of the methionine choline deficient (MCD) diet in C57BL/6J mice.

Serum (mg/L)	Normal	MCD	DMC	CM	BDMC
TC	33.1 ± 2.5 c	67.5 ± 2.5 a	42.5 ± 1.7 b	44.5 ± 2.1 b	40.5 ± 2.3 b
TG	51.2 ± 1.5 b	83.2 ± 8.7 a	58.2 ± 3.5 b	58.2 ± 3.7 b	53.6 ± 5.2 b
HDL	44.5 ± 2.7 a	29.5 ± 4.5 c	37.2 ± 3.2 b	33.2 ± 3.2 bc	32.5 ± 2.6 bc
LDL	8.8 ± 2.1 d	36.5 ± 2.5 a	26.1 ± 2.8 b	29.9 ± 3.1 b	13.7 ± 1.2 c

Data are expressed as mean ± SD of seven animals. Different letters in the same line represent significant differences (*p* < 0.05) following Duncan’s multiple range test. TG, triglyceride; TC, total cholesterol; HDL, high-density lipoprotein; LDL, low-density lipoprotein. DMC: demethoxycurcumin; CM: curcumin; BDMC: demethoxycurcumin.

## Data Availability

The data presented in this study are available on request from the corresponding author. The data are not publicly available due to ongoing longitudinal analysis.

## References

[1] Ahn E.M., Choi S.A., Choi J.Y. (2017). HPLC analytical method validation of Aralia elata extract as a functional ingredients. Korean J. Food Preserv..

[2] Song S.H., Choi J.A. (2010). A Study on the Trend of World Traditional Medicine and Key Area of Traditional Korean Medicine(TKM) R&D. Korean J. Orient. Med..

[3] Keum J.H., Han H.Y., Seok J.H., Roh H.S., Lee J.K., Jeong J.Y., Kim J.A., Woo M.H., Choi J.S., Min B.S. (2014). Analysis and Stability Test of the Extracts from Epimedii Herba, Atractylodis Rhizoma Alba and Polygalae Radix for Toxicity Study. Korean J. Pharmacogn..

[4] Kim S.G., Sharma D.K., Ramakanta L., Lee K.H., Han S.M., Jeong H.J. (2014). Development of Analytical Method for Quality Control from New Herbal Medicine(HPL-4). Korean J. Pharmacogn..

[5] Lee M.K., Lee J.S., Kwack S.J., Kim J.M., Kang T.S., Lee J.H., Woo M.H., Choi J.S., Bae K.H., Min B.S. (2009). Analysis and Stability test of the Extract of Coptidis Rhizoma and Salviae Miltiorrhizae Radix for Toxicity Study. Korean J. Pharmacogn..

[6] Jeon S.Y., Jeong E.J., Baek J.H., Cha Y.J. (2011). Analytical Method Validation of Quercetin in Changnyeong Onion Extract as a Functional Ingredient for Functional Health Food. J. Korean. Soc. Food Sci Nutr..

[7] Chae K.S., Son R.H., Park S.Y., Kim K.A., Lee T.B., Kwon J.W. (2014). Analytical Method Validation of Ellagic Acid as a Marker Compound for the Standardization of Black Raspberry Extract as a Functional Ingredient. Food Eng. Prog..

[8] Pang Y., Kartsonaki C., Turnbull I., Guo Y., Chen Y., Clarke R., Bian Z., Bragg F., Millwood I.Y., Yang L. (2019). Adiposity in relation to risks of fatty liver, cirrhosis and liver cancer: A prospective study of 0.5 million Chinese adults. Sci. Rep..

[9] Bijnen M., van Greevenbroek M.M.J., van der Kallen C.J.H., Scheijen J.L., van de Waarenburg M.P.H., Stehouwer C.D.A., Wouters K., Schalkwijk C.G. (2019). Hepatic Fat Content and Liver Enzymes Are Associated with Circulating Free and Protein-Bound Advanced Glycation End Products, Which Are Associated with Low-Grade Inflammation: The CODAM Study. J. Diabetes Res..

[10] Al-Muzafar H.M., Amin K.A. (2018). Thiazolidinedione induces a therapeutic effect on hepatosteatosis by regulating stearoyl-CoA desaturase-1, lipase activity, leptin and resistin. Exp. Ther. Med..

[11] Hoang S.A., Oseini A., Feaver R.E., Cole B.K., Asgharpour A., Vincent R., Siddiqui M., Lawson M.J., Day N.C., Taylor J.M. (2019). Gene Expression Predicts Histological Severity and Reveals Distinct Molecular Profiles of Nonalcoholic Fatty Liver Disease. Sci. Rep..

[12] Weingarten T.N., Mantilla C.B., Swain J.M., Kendrick M.L., Oberhansley J.M., Burcham R.J., Ribeiro T.C., Watt K.D., Schroeder D.R., Narr B.J. (2012). Nonalcoholic steatohepatitis in bariatric patients with a diagnosis of obstructive sleep apnea. Obes. Facts.

[13] Gholami A., Zamani F., Hosseini B., Sharafkhani R., Maadi M., Moosavi Jahromi Z., Khazaee-Pool M., Sohrabi M. (2018). Metabolic Syndrome Is Associated with Health-Related Quality of Life in Suspected Patients with Nonalcoholic Steatohepatitis. Med. Princ. Pract..

[14] Uchida D., Takaki A., Adachi T., Okada H. (2018). Beneficial and Paradoxical Roles of Anti-Oxidative Nutritional Support for Non-Alcoholic Fatty Liver Disease. Nutrients.

[15] Lonardo A., Mantovani A., Lugari S., Targher G. (2019). NAFLD in Some Common Endocrine Diseases: Prevalence, Pathophysiology, and Principles of Diagnosis and Management. Int. J. Mol. Sci..

[16] Liao Y.Y., Yeh C.K., Huang K.C., Tsui P.H., Yang K.C. (2019). Metabolic Characteristics of a Novel Ultrasound Quantitative Diagnostic Index for Nonalcoholic Fatty Liver Disease. Sci. Rep..

[17] Lee Y., Kwon E.Y., Choi M.S. (2018). Dietary Isoliquiritigenin at a Low Dose Ameliorates Insulin Resistance and NAFLD in Diet-Induced Obesity in C57BL/6J Mice. Int. J. Mol. Sci..

[18] Li Y., Zhao X., Feng X., Liu X., Deng C., Hu C.H. (2016). Berberine Alleviates Olanzapine-Induced Adipogenesis via the AMPKalpha-SREBP Pathway in 3T3-L1 Cells. Int. J. Mol. Sci..

[19] Yokota S., Nakamura K., Ando M., Kamei H., Hakuno F., Takahashi S., Shibata S. (2014). Acetylcholinesterase (AChE) inhibition aggravates fasting-induced triglyceride accumulation in the mouse liver. FEBS Open Bio.

[20] Boudaba N., Marion A., Huet C., Pierre R., Viollet B., Foretz M. (2018). AMPK Re-Activation Suppresses Hepatic Steatosis but its Downregulation Does Not Promote Fatty Liver Development. EBioMedicine.

[21] Zhao D., Sun X., Lv S., Sun M., Guo H., Zhai Y., Wang Z., Dai P., Zheng L., Ye M. (2019). Salidroside attenuates oxidized lowdensity lipoproteininduced endothelial cell injury via promotion of the AMPK/SIRT1 pathway. Int. J. Mol. Med..

[22] Lin Z.H., Li Y.C., Wu S.J., Zheng C., Lin Y.Z., Lian H., Lin W.Q., Lin J.F. (2019). Eliciting alpha7-nAChR exerts cardioprotective effects on ischemic cardiomyopathy via activation of AMPK signalling. J. Cell. Mol. Med..

[23] Zhang R., Chen J., Mao X., Qi P., Zhang X. (2019). Separation and Lipid Inhibition Effects of a Novel Decapeptide from Chlorella pyenoidose. Molecules.

[24] Zheleva-Dimitrova D. (2013). Antioxidant and acetylcholinesterase inhibition properties of *Amorpha fruticosa* L. and *Phytolacca americana* L.. Pharmacogn. Mag..

[25] Abdou E.M., Fayed M.A.A., Helal D., Ahmed K.A. (2019). Assessment of the hepatoprotective effect of developed lipid-polymer hybrid nanoparticles (LPHNPs) encapsulating naturally extracted beta-Sitosterol against CCl4 induced hepatotoxicity in rats. Sci. Rep..

[26] Sun J., Jiang T., Xu W., Feng Z., Quan X., Leng P., Sun W., Zhao J., Jing F., Li J. (2019). Quantification of 1D, a novel derivative of curcumin with potential antitumor activity, in rat plasma by liquid chromatography-tandem mass spectrometry: Application to a pharmacokinetic study in rats. Pharm. Biol..

[27] Anandakumar S., Joseph J.A., Bethapudi B., Agarwal A., Jung E.B. (2014). Anti-inflammatory Effects of Turmeric (*Curcuma longa* L.) Extract on Acute and Chronic Inflammation Models. J. Korean. Soc. Food Sci. Nutr..

[28] Jeong H.J., Kim S.T., Park J.J., Kim K.H., Kim K.M., Jun W.J. (2017). Antioxidant Activities and Protective Effects of Hot Water Extract from *Curcuma longa* L. on Oxidative Stress-Induced C2C12 Myoblasts. J. Korean. Soc. Food Sci. Nutr..

[29] Kang W.S., Kim J.H., Park E.J., Yoon K.R. (1998). Antioxidative property of turmeric (Curcuma Rhizoma) ethanol extract. Korean J. Food Sci. Technol..

[30] Mapoung S., Suzuki S., Fuji S., Naiki-Ito A., Kato H., Yodkeeree S., Ovatlarnporn C., Takahashi S., Limtrakul Dejkriengkraikul P. (2019). Cyclohexanone curcumin analogs inhibit the progression of castration-resistant prostate cancer in vitro and in vivo. Cancer Sci..

[31] Hay E., Lucariello A., Contieri M., Esposito T., De Luca A., Guerra G., Perna A. (2019). Therapeutic effects of turmeric in several diseases: An overview. Chem. Biol. Interact..

[32] Li S., Yuan W., Deng G., Wang P., Yang P. (2011). Chemical composition and product quality control of turmeric (*Curcuma longa* L.). Pharm. Crops..

[33] European Medicines Agency (2016). Committee on Herbal Medicinal Products; Assessment Report on Curcuma longa L. rhizoma.

[34] Pfeiffer E., Hohle S., Solyom A.M., Metzler M. (2003). Studies on the stability of turmeric constituents. J. Food Eng..

[35] Haudi S., Artanti A.N., Rinanto Y., Wahyuni D.S.C. (2018). Curcuminoid content of *Curcuma longa* L. and Curcuma xanthorrhiza rhizome based on drying method with NMR and HPLC-UVD. Mater. Sci. Eng..

[36] Ruby A.J., Kuttan G., Dinesh Babu K., Rajasekharan K.N., Kuttan R. (2018). Anti-tumour and antioxidant activity of natural curcuminoids. Mater. Sci. Eng..

[37] Zhong Y., Yu W., Feng J., Fan Z., Li J. (2014). Curcumin suppresses tumor necrosis factor-alpha-induced matrix metalloproteinase-2 expression and activity in rat vascular smooth muscle cells via the NF-kappaB pathway. Exp. Ther. Med..

[38] Ding T., Li T., Wang Z., Li J. (2017). Curcumin liposomes interfere with quorum sensing system of Aeromonas sobria and in silico analysis. Sci. Rep..

[39] Lin Y.L., Tsai N.M., Chen C.H., Liu Y.K., Lee C.J., Chan Y.L., Wang Y.S., Chang Y.C., Lin C.H., Huang T.H. (2019). Specific drug delivery efficiently induced human breast tumor regression using a lipoplex by non-covalent association with anti-tumor antibodies. J. Nanobiotechnol..

[40] Alam S., Panda J.J., Mukherjee T.K., Chauhan V.S. (2016). Short peptide based nanotubes capable of effective curcumin delivery for treating drug resistant malaria. J. Nanobiotechnol..

[41] Hatamipour M., Ramezani M., Tabassi S.A.S., Johnston T.P., Sahebkar A. (2019). Demethoxycurcumin: A naturally occurring curcumin analogue for treating non-cancerous diseases. J. Cell. Physiol..

[42] Hatamipour M., Ramezani M., Tabassi S.A.S., Johnston T.P., Ramezani M., Sahebkar A. (2018). Demethoxycurcumin: A naturally occurring curcumin analogue with antitumor properties. J. Cell. Physiol..

[43] Du Z., Sha X. (2017). Demethoxycurcumin inhibited human epithelia ovarian cancer cells’ growth via up-regulating miR-551a. Tumour Biol..

[44] Xiang M., Jiang H.G., Shu Y., Chen Y.J., Jin J., Zhu Y.M., Li M.Y., Wu J.N., Li J. (2020). Bisdemethoxycurcumin Enhances the Sensitivity of Non-small Cell Lung Cancer Cells to Icotinib via Dual Induction of Autophagy and Apoptosis. Int. J. Biol. Sci..

[45] Kim S.B., Kang O.H., Lee Y.S., Han S.H., Ahn Y.S., Cha S.W., Seo Y.S., Kong R., Kwon D.Y. (2016). Hepatoprotective Effect and Synergism of Bisdemethoycurcumin against MCD Diet-Induced Nonalcoholic Fatty Liver Disease in Mice. PLoS ONE.

[46] Ramezani M., Hatamipour M., Sahebkar A. (2018). Promising anti-tumor properties of bisdemethoxycurcumin: A naturally occurring curcumin analogue. J. Cell. Physiol..

[47] Zhang J., Han H., Shen M., Zhang L., Wang T. (2019). Comparative Studies on the Antioxidant Profiles of Curcumin and Bisdemethoxycurcumin in Erythrocytes and Broiler Chickens. Animals.

[48] Akbar A., Kuanar A., Joshi R.K., Sandeep I.S., Mohanty S., Naik P.K., Mishra A., Nayak S. (2016). Development of Prediction Model and Experimental Validation in Predicting the Curcumin Content of Turmeric (*Curcuma longa* L.). Front. Plant Sci..

[49] Yan C., Zhang Y., Zhang X., Aa J., Wang G., Xie Y. (2018). Curcumin regulates endogenous and exogenous metabolism via Nrf2-FXR-LXR pathway in NAFLD mice. Biomed. Pharmacother..

[50] Jeong S.C., Kim S.M., Jeong Y.T., Song C.H. (2013). Hepatoprotective effect of water extract from *Chrysanthemum indicum* L. flower. Chin. Med..

[51] Zhou D., Fan J.G. (2019). Microbial metabolites in non-alcoholic fatty liver disease. World J. Gastroenterol..

[52] Del Campo J.A., Gallego-Duran R., Gallego P., Grande L. (2018). Genetic and Epigenetic Regulation in Nonalcoholic Fatty Liver Disease (NAFLD). Int. J. Mol. Sci..

[53] Ahmad O., Wang B., Ma K., Deng Y., Li M., Yang L., Yang Y., Zhao J., Cheng L., Zhou Q. (2019). Lipid Modulating Anti-oxidant Stress Activity of Gastrodin on Nonalcoholic Fatty Liver Disease Larval Zebrafish Model. Int. J. Mol. Sci..

[54] Muangnoi C., Ratnatilaka Na Bhuket P., Jithavech P., Supasena W., Paraoan L., Patumraj S., Rojsitthisak P. (2019). Curcumin diethyl disuccinate, a prodrug of curcumin, enhances anti-proliferative effect of curcumin against HepG2 cells via apoptosis induction. Sci. Rep..

[55] Guevara-Flores A., Martinez-Gonzalez J.J., Herrera-Juarez A.M., Rendon J.L., Gonzalez-Andrade M., Torres Duran P.V., Enriquez-Habib R.G., Del Arenal Mena I.P. (2019). Effect of curcuminoids and curcumin derivate products on thioredoxin-glutathione reductase from Taenia crassiceps cysticerci. Evidence suggesting a curcumin oxidation product as a suitable inhibitor. PLoS ONE.

[56] Lee Y.S., Lee D.Y., Kwon D.Y., Kang O.H. (2020). Improvement Effect of Non-alcoholic Fatty Liver Disease by *Curcuma longa* L. Extract. Korean J. Med. Crop. Sci..

[57] Lu C., Zhang F., Xu W., Wu X., Lian N., Jin H., Chen Q., Chen L., Shao J., Wu L. (2015). Curcumin attenuates ethanol-induced hepatic steatosis through modulating Nrf2/FXR signaling in hepatocytes. IUBMB Life.

[58] Oguz A., Kapan M., Onder A., Kilic E., Gumus M., Basarali M.K., Firat U., Boyuk A., Buyukbas S. (2013). The Effects of Curcumin on the Liver and Remote Organs after Hepatic Ischemia Reperfusion Injury Formed With Pringle Manoeuvre in Rats. Eur. Rev. Med. Pharmacol. Sci..

[59] Chen Y.Y., Lin Y.J., Huang W.T., Hung C.C., Lin H.Y., Tu Y.C., Liu D.M., Lan S.J., Sheu M.J. (2018). Demethoxycurcumin-Loaded Chitosan Nanoparticle Downregulates DNA Repair Pathway to Improve Cisplatin-Induced Apoptosis in Non-Small Cell Lung Cancer. Molecules.

[60] Cho J., Lee I., Kim D., Koh Y., Kong J., Lee S., Kang H. (2014). Effect of aerobic exercise training on non-alcoholic fatty liver disease induced by a high fat diet in C57BL/6 mice. J. Exerc. Nutr. Biochem..

[61] Pela´ez N., Gavalda-Miralles A., Wang B., Navarro H.T., Gudjonson H., Rebay I., Dinner A.R., Katsaggelos A.K., Amaral L.N., Carthew R.W. (2015). Dynamics and Heterogeneity of a Fate Determinant during Transition towards Cell Differentiation. Elife.

[62] Hardie D.G. (2007). AMP-activated/SNF1 protein kinases: Conserved guardians of cellular energy. Nat. Rev. Mol. Cell Biol..

[63] Chen Y.C., Chen H.J., Huang B.M., Chen Y.C., Chang C.F. (2019). Polyphenol-Rich Extracts from Toona sinensis Bark and Fruit Ameliorate Free Fatty Acid-Induced Lipogenesis through AMPK and LC3 Pathways. J. Clin. Med..

[64] Botezelli J.D., Coope A., Ghezzi A.C., Cambri L.T., Moura L.P., Scariot P.P., Gaspar R.S., Mekary R.A., Ropelle E.R., Pauli J.R. (2016). Strength Training Prevents Hyperinsulinemia, Insulin Resistance, and Inflammation Independent of Weight Loss in Fructose-Fed Animals. Sci. Rep..

[65] Yu J., Liu X., Li Y., Meng S., Wu F., Yan B., Xue Y., Ma T., Yang J., Liu J. (2018). Maternal exposure to farming environment protects offspring against allergic diseases by modulating the neonatal TLR-Tregs-Th axis. Clin. Transl. Allergy..

[66] Tosello-Trampont A.C., Landes S.G., Nguyen V., Novobrantseva T.I., Hahn Y.S. (2012). Kuppfer cells trigger nonalcoholic steatohepatitis development in diet-induced mouse model through tumor necrosis factor-alpha production. J. Biol. Chem..

